# The contribution of astrocytes to obesity-associated metabolic disturbances

**DOI:** 10.7555/JBR.36.20200020

**Published:** 2022-08-28

**Authors:** Marta Obara-Michlewska

**Affiliations:** Department of Neurotoxicology, Mossakowski Medical Research Institute, Polish Academy of Sciences, Warsaw 02-106, Poland

**Keywords:** astrocytes, astrocytosis, hypothalamic inflammation, obesity, brain energy metabolism

## Abstract

Obesity is a worldwide health, economic and social concern, despite efforts made to counteract the spreading wave of eating and nourishment-associated disorders. The review aims to show how the glial cells, astrocytes, contribute to the central regulation of appetite and energy metabolism. The hypothalamus is the brain center responsible for nutrients and nutritional hormone sensing, signal processing, and execution of metabolic and behavioral responses, directed at sustaining energy homeostasis. The astrocytes are endowed with receptors, transporters and enzymatic machinery responsible for glucose, lactate, fatty acids, ketone bodies, as well as leptin or ghrelin transport and metabolism, and that render them supporters and partners for neurons in governing the brain and body energy intake and expenditure. However, the role of astrocytes associated with brain energy metabolism reaches far beyond simple fuel contingent—they contribute to cognitive performance. The cognitive decline which often accompanies high fat- and/or high-calorie diets and correlates with neuroinflammation and astrogliosis, is a major concern. The last two decades of research enabled us to acknowledge the astroglia in obesity-associated dysfunctions and to investigate astrocytes as contributors to the pathology, as well as targets for therapy.

## Introduction

Obesity is defined by the World Health Organisation (WHO) as an abnormal fat tissue accumulation that may cause health impairment, with the body mass index (BMI) ≥30 accepted as the threshold value. Obesity is regarded as one of the civilization diseases, next to diabetes, certain cancers, coronary disease, chronic obstructive pulmonary disease, or depression. According to the 2016 WHO report, since 1972 there has been a threefold increase in the worldwide overweight and obesity (BMI≥30) prevalence, reaching 39%. The European Statistical Office provided data from 2019, showing that 53% of European Union citizens have a BMI of 25 or higher. The analysis of 15 dietary factors in 195 countries in the period 1990–2017 revealed that one in five deaths is related to a poor diet, which exceeds the death toll from cigarette smoking^[1]^, well justifying the term 'obesity pandemic'. Obesity increases the risk of type 2 diabetes, non-alcoholic fatty liver disease, cardiovascular disease, Alzheimer's disease, depression, and breast, ovary, liver and colon cancer, shortening the life expectancy substantially. It decreases the life quality not only due to health deterioration but also due to social problems, like unemployment. Obesity is also a burden from an economic point of view. The level of fat tissue, seemingly simply increasing due to the higher energy intake than expenditure, is a result of the complex interaction of heterogeneous environmental and genetic predispositions, affecting eating behavior, individual energy expenditure, and physical activity. The genetic background has been recognized as an important, 47%–80% risk factor in obesity^[2–3]^. More than a hundred genes linked with obesity were identified^[4–5]^. The most common is the polygenic predisposition to excessive weight gain, whereas monogenic obesity accounts for up to 5% of cases only, and results mainly from mutations in genes involved in the pro-opiomelanocortin pathway in the brain^[4,6]^. Despite social programs and research efforts proportional to the scale of the obesity-related health impoverishment, effective, safe, and accessible prevention or treatment is still lacking. Therefore, research on both psychological and molecular mechanisms underlying overweight or obesity is needed.

This review provides a summary of brain energy metabolism and regulation of appetite and feeding by the central nervous system (CNS), with a focus on the role of astrocytes. Their involvement in metabolic disturbances underlying obesity has been increasingly acknowledged, and growing evidence emerges on the importance of astrocyte-mediated signaling and energy metabolism homeostasis in keeping the appropriate proportions of the adipose tissue. For the involvement of other types of glial cells, especially microglia, see *e.g.* the extensive review by Argente-Arizón *et al*^[7]^.

Astrocytes are multitasked glial cells, no longer recognized as passive neurons' supporters. Astrocytes constitute the blood brain barrier (BBB), regulating blood flow, as well as transporting a plethora of compounds, including nutrients. They regulate ionic homeostasis of interstitial fluid— tightly regulate pH and K^+^, ensuring proper enzymatic and neuronal activity. As astrocytes express the majority of glutamate transporters, they also assert the proper conditions for neurotransmission and neuronal excitability by uptaking glutamate from the synaptic cleft and concurrently protect the CNS from excitotoxicity. They also protect neurons from oxidative/nitrosative stress, due to the high level of expression and activity of antioxidative enzymes. Moreover, astrocytes are an immanent element of the glymphatic system, responsible for the clearance of unnecessary or potentially harmful metabolites^[8]^. Astrocytes take part in the glutamine–glutamate cycle, synthesizing glutamine from glutamate and ammonia— the newly formed glutamine is then uptaken by neurons and used as a precursor for glutamate and gamma-aminobutyric acid (GABA) synthesis. Astrocytes are active partners in the tripartite synapse, by releasing the gliotransmitters such as adenosine triphosphate (ATP), adenosine, and D-serine. Astrocytes also release pro- and anti-inflammatory cytokines, as well as trophic factors, *e.g.*, brain-derived neurotrophic factor. By doing all these, astrocytes regulate synaptic transmission, synaptogenesis, neuronal differentiation and proliferation, and modulate molecular pathways underlying cognitive functions and behavior^[5,9–10]^. Here, the focus will be on astrocytes' ability to recognize nutrients or nutritional hormones and supply the neurons with energy metabolites, such as glucose, lactate or ketone bodies.

## The role of the hypothalamus in the control of food intake

Food intake and energy expenditure are controlled and regulated at the CNS level. The hypothalamus is the brain region where the orexigenic (appetite-increasing, anabolic) and anorexigenic (appetite-suppressing, catabolic) signals are received and processed. Nutrients, such as glucose or fatty acids, and hormones, *e.g.*, leptin or ghrelin, may serve as such signals.

The orexigenic and anorexigenic input required by the hypothalamus to adequately respond to the energy status and to adjust the feeding behavior, involve mechanical, thermal, metabolic and hormonal signals. The mechanical signal of satiety is sent through the vagus nerve from the mechanoreceptors in the stomach when its walls are stretched by food^[11]^. The peripheral signals exerting an anorexigenic effect are insulin and leptin, whereas ghrelin is the orexigenic one. The signals that originate in the CNS cells include neuropeptide Y (NPY), agouti-related peptide (AgRP), α-melanocyte-stimulating hormone (α-MSH), corticotropin- and thyrotropin-releasing hormones (CRH and TRH, respectively), and the first three are orexigenic and the latter anorexigenic^[12–13]^.

The ventromedial nucleus (VMN) of the hypothalamus is called the satiety center, whereas the lateral hypothalamus (LH) is the hunger center, as lesions of those areas lead to obesity or starvation, respectively^[14]^. In the arcuate nucleus (ARC) of the hypothalamus, there are two populations of neurons: the anorexigenic pro-opiomelanocortin (POMC) neurons and orexigenic NPY/AgRP neurons. Both POMC and NPY/AgRP neurons receive signals from other brain regions and the peripheral organs. The nutritional and hormonal signals from the periphery can reach the ARC neurons because the BBB becomes less tight and selective in the median eminence of the hypothalamus, an area with fenestrated capillaries, located in the proximity of the ARC^[15]^. The POMC neurons project mainly to the paraventricular hypothalamic nucleus (PVN) and also to the dorsomedial (DMN) nucleus, LH, and VMN. Those hypothalamic nuclei integrate signals and transmit them to the extrahypothalamic structures, to exert a response balancing the energy intake and expenditure by adequate metabolism and feeding behavior.

The POMC neurons, receiving nutrient or hormonal signals, release α-MSH, a product of POMC cleavage that activates melanocortin 3 and 4 receptors (MC3/4R), which are expressed throughout the brain, but mostly in the PVN^[16]^, and the activation of the neurons results in the inhibition of food intake and increased energy expenditure (similarly to VMN, destruction of PVN leads to obesity). Conversely, lack of nutrients is a signal for NPY/AgRP neurons, projecting to LH and PVN. The NPY drives feeding through activation of receptors Y1, Y2, Y4, Y5, as well as Y6 in mice^[17]^, located widely in the brain^[18]^. The anorexigenic signaling is under the feedback control from the NPY/AgRP neurons. The NPY, acting *via* Y1 receptors, inhibits POMC neurons, whilst *via* Y1 or Y5 activates other neurons, responsible for executing feeding. The activation also involves the inhibitory neurons and release of GABA which additionally inhibits POMC neurons. The AgRP is an inverse agonist of MC4R and by blocking the action of α-MSH, inhibits the anorexigenic signaling^[17]^.

In the POMC neurons of ARC, there is also the cocaine- and amphetamine-regulated transcript (CART) expressed. Its co-expression with α-MSH reaches 100%. The CART neuropeptide is also involved in the control of the reward and addiction system, and appetite and body weight. High levels of CART, historically found overexpressed in cocaine abusers, are present not only in the hypothalamus (ARC and LH) but also in the PVN or nucleus accumbens, the brain regions engaged in appetite control and energy expenditure. The CART effects depend on which hypothalamic nuclei are involved— LH CART was reported to stimulate appetite, whereas ARC CART had the opposite effect^[19]^. Although CART is present both in the CNS and in the periphery, the receptors or agonists for CART have not been identified^[20]^.

**Figure 1:**
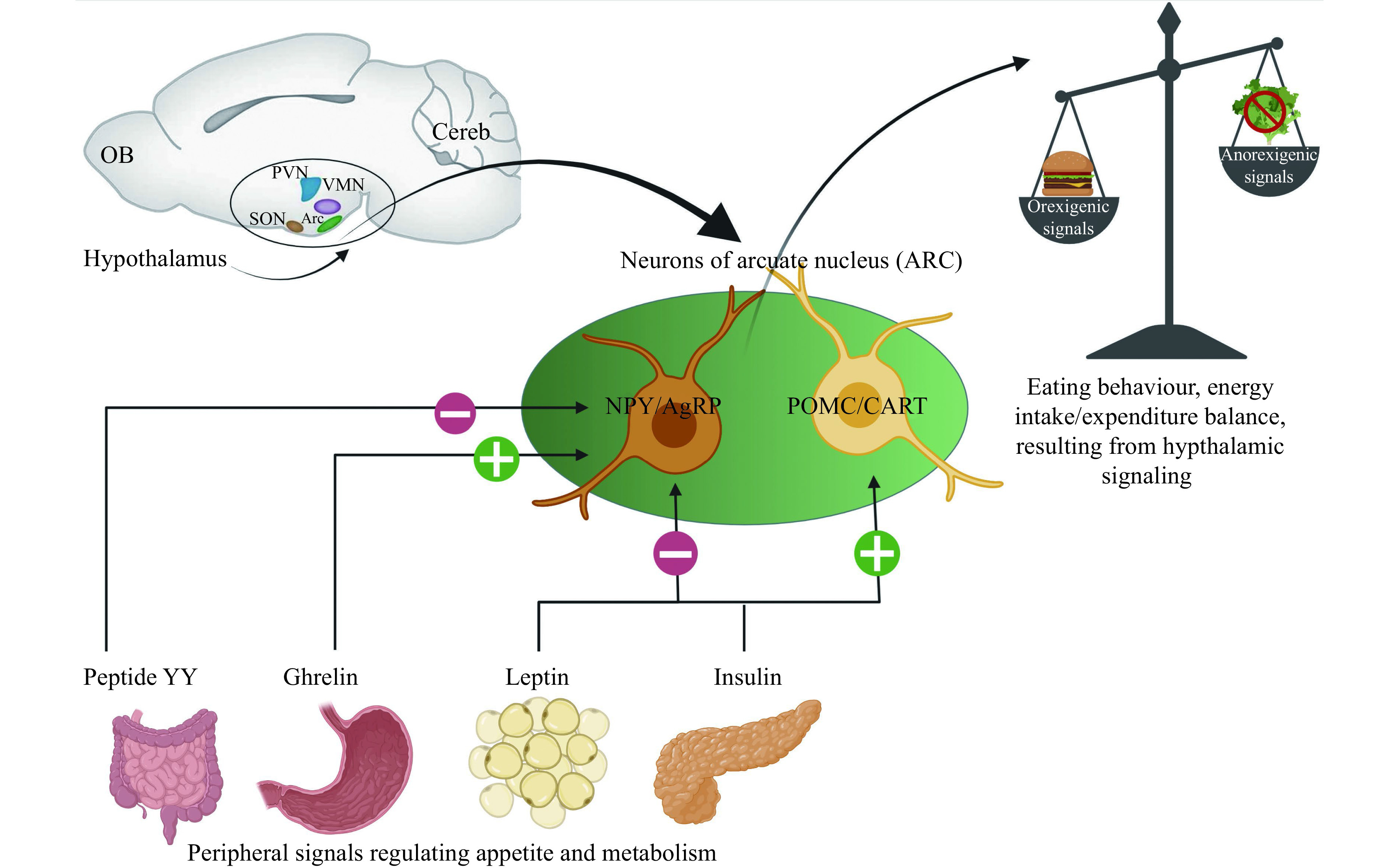
The simplified scheme of the hypothalamus-based control over appetite.

The hypothalamus-based control over appetite, processing major signals from the periphery, is schematically depicted in ***Fig. 1***.

## Astrocytic contribution to the brain energy metabolism

The brain is an organ with exceptionally high demand for energy. Its major source for neurons is glucose, but lactate, fatty acids or ketone bodies may also be utilized in specific conditions. Here the basic role of astrocytes emerges as energy-supporters for neurons, because there mainly in astrocytes are expressed or activated enzymes enabling metabolism of lactate, fatty acids, ketones, or glycogen^[10]^. That, and also the basic fact that astrocytes are equipped with glucose transporters and insulin receptors, render them partners of neurons in governing the brain energy metabolism and feeding behavior.

### Glucose

Astrocytes constitute an integral, and functional element of the BBB. Their endfeet almost entirely cover the surface of the capillaries, thus the blood-borne glucose reaches the energetically highly demanding neurons mostly *via* astrocytes— only a minority of glucose present in the extracellular fluid is derived directly from blood, bypassing astrocytes. Moreover, astrocytes release molecules, such as epoxyeicosatrienoic acid, prostaglandin E2, K^+^ or ATP that can modulate the local blood flow^[21]^, adjusting the nutrient supply. Therefore, astrocytes actively contribute to the regulation of glucose levels in the brain and, consequently, in the peripheral tissues.

Glucose is the major energy source for the brain and astrocytes secure both glucose transport and storage. Astrocytes are endowed primarily with the glucose transport protein type 1 (GLUT1) glucose transporter, whereas neurons express mostly the GLUT3^[22]^. The GLUT1, which abundantly presents also in endothelial cells, facilitates bidirectional glucose transport across the cell membranes, whereas GLUT3 is responsible for the glucose uptake^[22]^.

The GLUT1 expression was found to be decreased in hypothalamic astrocytes of hyperglycaemic rats, with streptozotocin-induced diabetes. The lack of the glial GLUT1 prevented the increase of the glucose level in the hypothalamus, disrupting the negative feedback control loop and resulting in disability to reduce glucose in the peripheral blood circulation. The overexpression of GLUT1 in astrocytes, by means of adenoviral transfection, normalized the plasma glucose levels in diabetic rats^[23]^. Another astroglial gap-junctions forming protein, connexin 43 (Cx43), was shown to be required for glucose sensing in the hypothalamus. Cx30 and Cx43 are expressed at a relatively high level in the mediobasal hypothalamus (MBH) and both hyperglycemia and hypoglycemia modulate their expression. The selective downregulation of Cx43 in the MBH by siRNA transfection resulted in the loss of glucose sensitivity in the hypothalamus, which was demonstrated by the attenuated insulin release response to intracarotid glucose bolus administration in the rats with Cx43 downregulation^[24]^.

The glucose uptaken by astrocytes may be metabolized to glycogen or lactate. In fact, in astrocytes, most glucose is metabolized *via* the glycolytic pathway, whereas in neurons *via* the oxidative one^[25]^. First, glucose is phosphorylated by hexokinase to glucose-6-phosphate. Then, it may either be metabolized to glycogen by glycogen synthase or go through the glycolytic pathway, and pyruvate and lactate may be synthesized by pyruvate kinase and lactate dehydrogenase, respectively. The glycogen-stored glucose may be mobilized and catalyzed back to glucose by glycogen phosphorylase. The neurons are not in possession of active glycogen synthase, therefore a neuron-astrocyte lactate shuttle model has been proposed^[26]^, where astrocytes, reacting to signals from neurons, release lactate *via* monocarboxylate transporters 1 and 4 (MCT1, MCT4). Lactate serves as a fuel for neurons in a situation of increased demand (increased neuronal activity, and decreased glucose supply), to protect neurons from hypoglycemia^[27]^. It is still debatable, however, whether astrocytes release glucose that may be utilized by neurons as a fuel^[26,28]^. Moreover, lactate released by astrocytes may not only be used as energy but also as a signaling molecule in synaptic plasticity, memory, or drug addiction^[29]^. The pharmacological or genetically-engineered inhibition of MCT1/2 results in the impairment of long-term memory and memory consolidation/reconsolidation in mice^[25]^. It was shown that specifically orexigenic neurons of ARC are sensitive to the astrocyte-derived lactate signal, as revealed by the electrophysiological recordings, employing the MCT blocker, α-cyano-4-hydroxycinnamate, and fluoroacetate, a selective astroglial toxin^[30]^.

The glycolysis conducted by astrocytes is enhanced by neuronal activity, manifested by glutamate and noradrenaline increase in the synaptic cleft. The glutamate uptake induces also lactate synthesis and shuttles it to neurons. The state of alertness, awareness, and attention requires energy, which is ensured by noradrenaline, released by the locus coeruleus in the brainstem. The adrenergic receptors, both α and β subtypes, are expressed by astrocytes. To secure the energy for the neuronal activity, the uptake of glutamate increases also glycogen synthesis in astrocytes. The glycogenolysis is initiated by the noradrenaline signaling as well, both in normal and in a pathological situation of energy substrates depletion, *e.g.*, during sleep deprivation. The glycogen dynamic homeostasis, regulated *i.a.* by the glutamate and noradrenaline signaling, is required for memory formation and consolidation^[31]^.

### Insulin

Insulin, released by the pancreatic β-cells in response to increased glucose levels, is responsible for glucose metabolism in the peripheral tissues, where it increases glucose uptake. As the POMC and AgRP/NPY neurons express insulin receptors, insulin is also regulating energy homeostasis at the central level. The insulin-mediated activation of AgRP/NPY neurons results in their inhibition, eventually leading to interleukin 6 (IL-6)-dependent inhibition of glucose-6-phosphatase and decreased glucose synthesis^[15]^. Activation of insulin receptors at POMC neurons results in their inhibition rather than activation, although an activatory effect has also been reported^[15,32]^. However, the experimental deprivation of POMC neurons of insulin receptors does not affect glucose or energy metabolism^[15]^.

The traditional view that, unlike in other tissues, the brain glucose uptake is not increased by insulin^[33]^, has been challenged^[33]^. Although most neurons and astrocytes are equipped with insulin-independent GLUT3 and GLUT1 glucose transporters, respectively, there has been discovered that the GLUT4 receptors in the hippocampus are translocated to the membrane in response to the insulin signal^[34]^.

The insulin receptor occurs in two isoforms: IR-B (long) and IR-A (short), generated by alternative splicing. The IR-B is ubiquitous in the peripheral tissues, whereas in the brain both isoforms are present, and IR-A is more prominent. The neurons express IR-A only, while astrocytes express both IR-A and IR-B^[35]^. The insulin binding results in autophosphorylation and consequent activation of the receptor. The insulin receptors may also be activated by the insulin-like growth factors (IGF) 1 and 2. Moreover, insulin may regulate astrocytic glucose metabolism by cooperating with IGF-1^[36]^. In astrocytes *in vitro*, decreasing IGF-1 receptor resulted in the impairment of mitochondrial function and ATP synthesis and overproduction of reactive oxygen species (ROS)^[37–38]^. Of note, by constructing transgenic mice overexpressing mitochondrial catalase, Vicente *et al* proved that decreased ROS production, modulated by glucose utilization *via* the pentose-phosphate pathway and increased glutathione metabolism, affects brain redox, carbohydrate, lipid and amino acid metabolism, as well as neuronal function and behavior^[39]^. Astrocyte-specific knock-out of IGF-1 receptor disturbs hippocampal-dependent spatial learning^[37]^.

Similarly, mice with simultaneous neuronal and astrocytic knock-out, as well as astrocyte-specific deletion of insulin receptors suffered from hypogonadism and delayed puberty^[40]^, as well as metabolic dysfunctions: increased appetite and obesity, and altered response to hypoglycemia^[41]^. Moreover, the astrocyte-specific knock-out mice presented increased anxiety- and depressive-like behavior. The knock-out decreased ATP release from astrocytes, resulting in purinergic signaling inhibition on dopaminergic neurons and, in consequence, decreased dopamine release from brain slices^[41]^.

### Lipids

The lipid homeostasis in the brain, an organ very rich in lipids^[42]^, a prerequisite for the myelination process, is sustained mostly by astrocytes^[33]^. The brain apolipoproteins, facilitating the transport of lipids (*i.a.* triacylglycerols, cholesterol, phospholipids) are synthesized mostly by astrocytes. In the brain, the most prevalent classes of apolipoproteins are E and J (ApoE and ApoJ, respectively)^[43–44]^. ApoE may also modulate the appetite: its intracerebral administration decreased food intake in rats and induced POMC expression^[45]^. The expression of ApoE was decreased in fasted rats and its level increased after food intake. Moreover, genetically obese mice with leptin deficiency (*ob/ob*) and rats with obesity induced by the high-fat diet had reduced levels of ApoE in the hypothalamus. Altogether, those results indicate that ApoE may serve as a satiety signal^[45]^.

The fatty acids, triacylglycerol (known also as triglyceride) derivatives, are the important fuel source for the brain cells and are also the satiety signal for the hypothalamic neurons. Both VMN neurons and astrocytes express fatty acid transporters, including FATP1, FATP4, and CD36. Ebrahimi *et al* showed that fatty acid binding protein 7 (FABP7), expressed in astrocytes and playing a role in fatty acid uptake, transport, and metabolism, is important for cognitive function^[46]^. Mice with FABP7 knock-out present abnormal dendritic morphology and excitatory synaptic function of cortical neurons^[46]^.

The excess of fatty acids generates a metabolic pathology, termed lipotoxicity. Treatment of human astrocytes *in vitro* with palmitate to induce lipotoxicity resulted in downregulation of seleno amino acids, glutamate and urea cycle metabolism. The increased level of alanine, adenosine and glutamine was proposed as a lipotoxicity marker^[47]^. When astrocytes in culture were chronically exposed to lipid emulsion, ectopic lipid storage was observable. The lipid droplets in cells were present long after the removal of lipids from the medium. As tested with insulin administration, lipotoxicity induced insulin resistance and disturbed glycogen synthesis. Moreover, the changed gene expression pattern of the hepatocyte growth factor signaling, IGF-1, insulin receptor, phosphoinositide 3-kinase/Akt kinase (PI3 K/AKT) and 5'-AMP-activated protein kinase (AMPK), was observed. The forkhead-box-protein O (FOXO) 1 and 3 were predicted as the upstream regulators^[48]^. The hypothalamic astrocytes treated with oleic acid or palmitic acid/triglycerides *ex vivo* responded with increased or decreased, respectively, expression of lipoprotein lipase (LPL) that hydrolyzes triglycerides in lipoproteins into free fatty acids. Nevertheless, postnatal knock-out of LPL resulted in greater body weight gain and glucose intolerance due to the high-fat diet, in comparison to the wild-type controls. Moreover, the LPL deficiency caused an increased accumulation of ceramide that might contribute to insulin resistance^[49]^. The astrocytes have a unique ability to produce ketone bodies by β-oxidation of fatty acids. Hypothalamic astrocytes have a higher enzymatic capacity for their β-oxidation than the cortical ones^[42]^. Both fatty acids and ketone bodies (mainly 3-β-hydroxybutyrate and acetoacetate) may serve as an energy source but the ketone bodies are produced and utilized in conditions of both hypoglycemia and high-fat diet. The ketone bodies may also cross the BBB through the endothelial MCT1 transporter and then being taken up by astrocytes, employing the MCT1 and MCT4 transporter, and also by neurons, almost exclusively employing MCT2^[50]^. When the level of fatty acids in the brain increases, as a result of a high-fat diet, astrocytes start their β-oxidation and the level of ketone bodies increases. In a situation when obese patients are starved to lose weight, as much as 60% of the energy utilized by the brain is covered by ketones^[25]^.

Since astrocytes are in control of lipid transport across BBB and within brain parenchyma, and of fatty acids and ketone bodies balance, they contribute to appetite control and metabolism regulation. Le Foll and Levin even proposed that the astrocyte-derived ketone bodies act in the hypothalamic neurons of VMN as an anorexigenic signal, which is stronger than the fatty acid translocase/receptor FAT/CD36-mediated fatty acids sensing, a mechanism employed by neurons^[51]^.

Cholesterol is essential for brain function, starting from membrane and vesicle continuity, myelin formation and synaptogenesis. In contrary to fatty acids, which are transported to the brain according to the concentration gradient, the cholesterol can not easily cross the BBB, and therefore is synthesized directly in the brain^[44]^, mostly by astrocytes. Ferris *et al* showed that cell-specific knock-out of the astrocytic transcription factor, sterol regulatory element binding protein 2 (SREBP2), responsible for the cholesterol synthesis, results in major disturbances in energy balance (*i.e.*, increased glucose oxidation by the brain) and body composition (*i.e.*, reduction in lean and fat mass)^[52]^. Moreover, SREBP2 is reduced in diabetic animals, which is accompanied by cognitive deficits^[53]^.

There are also studies indicating that astrocytes may be involved in the pathophysiology of obesity through interaction with peroxisome proliferator-activated receptors (PPARs). The PPARs are nuclear receptor proteins, with properties of transcription factors. They regulate cell differentiation and development as well as the metabolism of carbohydrates, proteins, and lipids. There are three groups of PPARs—α, δ/β, and γ—that are expressed in different tissues, including white and brown adipose tissue and brain. All three PPARs subtypes are present in the brain. In microglia and astrocytes, PPAR-γ predominates and regulates inflammation in the CNS^[54]^. The PPAR-γ is responsible for fatty acid storage and glucose metabolism, and PPAR-γ activated genes drive lipid uptake and adipogenesis. The brain PPAR-γ has been postulated to promote obesity in mice, as the PPAR-γ knock-out animals lack adipose tissue^[55]^.

In adipocytes or hepatocytes, the PPAR-γ is required for insulin sensitivity and mediates the insulin-sensitizing effects of anti-diabetic thiazolidinediones (TZDs), agonists of PPAR-γ. In mice with the neuron-specific knock-out of PPAR-γ, the high-fat diet resulted in decreased food intake and increased energy expenditure (thermogenesis), resulting in lesser weight gain. Moreover, the PPAR-γ knock-out animals responded better to leptin treatment. Concordantly, the effects of thiazolidinedione, *i.e.*, normalization of the glucose metabolism, were not present in knock-out mice^[55]^. Interestingly, mice with the PPAR-γ knock-out in astrocytes specifically, also had an obesity-predisposing phenotype but different comparing to the characteristic of the neuronal PPAR-γ knock-out mice^[56]^. The animals with astrocytic PPAR-γ deficiency presented a number of metabolic disturbances: decreased glucose tolerance and hepatic steatosis, and increased expression of genes involved in gluconeogenesis, lipid synthesis and transport. In addition, the astrocytic PPAR-γ deficient females had disturbances in sex hormones homeostasis^[56]^. The pioglitazone, another drug from the TZDs group, prevented depressive-like behavior in mice that were fed with high-fat diet for 12 weeks. The pioglitazone not only ameliorated the glucose metabolism disturbances, but also decreased astrogliosis, associated with obesity. The number of astrocytic branches was less decreased and glial fibrillary acidic protein (GFAP) immunoreactivity less pronounced after pioglitazone treatment^[57]^. The PPAR-γ agonists and TZDs were also proven to be effective in ameliorating cognitive deficits associated with Alzheimer's disease, both in pre-clinical studies and in the clinic^[54]^. However, the involvement of astrocytes in TZDs mechanism of action has not been investigated deep enough. On another note, also the PPAR-α agonists, fenofibrate and WY14643, were shown to be effective in inhibition of lipopolysaccharide (LPS) induced proinflammatory cytokine and nitric oxide release in primary mouse astrocytes^[58]^. Chistyakov *et al* showed that TLR agonists, inflammatory stimuli (*i.a.* LPS), resulted in the downregulation of PPARα/γ in rat astrocytes *in vitro*^[59]^.

### Leptin

Leptin, a metabolic hormone belonging to the family of adipokines, is released by adipocytes (white or brown) in amounts directly proportional to the mass of the fat tissue in the body. Leptin is called the satiety hormone, as it decreases energy expenditure and inhibits food intake. Leptin is a major regulator of the hypothalamic appetite-regulation related functions, by directly activating POMC/CART and inhibiting AgRP/NPY neurons^[60–61]^. The leptin exerts the anorexigenic/slimming effect by increased thermogenesis and energy expenditure, through the increasing activity of the sympathetic nervous system and norepinephrine turnover in the brown adipose tissue. The leptin-responsive POMC/CART neurons of hypothalamus project to sympathetic preganglionic neurons in the spinal cord^[61]^. In mice, six leptin receptor isoforms have been identified as products of alternative splicing or post-translational cleavage. The isoform b is responsible for the majority of leptin action. The mice that lack this isoform of the leptin receptors (*db/db* mice) present similar phenotypes to those with depletion of leptin ob/ob, namely hyperphagia, obesity, and hyperglycemia^[62]^. In the brain, leptin receptors are highly expressed in endothelial cells, astrocytes, and tanycytes^[28,63]^. The conditional, astrocyte-specific knock-out of leptin receptor leads to disturbances in astroglial morphology, and increases activation of both POMC and AgRP neurons. The mice with astrocytic leptin receptor deficiency responded weaker to leptin but stronger to ghrelin administration^[64]^. In obesity, leptin resistance is a common contributor. Analogically to insulin resistance, despite elevated blood leptin, it does not mediate its effects, in general due to the impairment either of leptin transport into the brain, or the central response^[28]^. The obesity-associated hyperleptinemia was shown to promote hypertension in rats through the mechanism depending on hypothalamic astroglial hypoxia-inducible factor 1α-vascular endothelial growth factor (HIF1α-VEGF)^[65]^, and not *via* the neuronal (pro)renin receptor, a component of the brain renin-angiotensin system^[66]^.

The increased levels of leptin were also found in the serum of rats with congenital hypothyroidism. The young rats presented a panoply of glutamatergic neurotransmission abnormalities, including decreased glutamine synthetase (GS) activity and increased glutamine accumulation in the cerebral cortex^[67]^. Of note, astrocytes play a central role in the thyroid hormone metabolism in the brain. They are majorly responsible for the uptake of the thyroxine (T4) from the circulation *via* OATP1C1 transporter and conversion to active 3,3′,5-triiodothyronine (T3) by the action of type 2 iodothyronine deiodinase, almost entirely expressed in glial cells^[68]^. The term 'glioendocrine' is used to describe this relationship between the endocrine system and the CNS represented by astrocytes^[69]^.

### Ghrelin

The hypothalamus receives hormonal signals from the periphery, including the gastrointestinal tract. Ghrelin is an orexigenic hormone released by an empty stomach, a signal that starvation lasts too long. Ghrelin activates AgRP/NP neurons, which results in increased appetite and eating. The intestinal hormones, glucagon-like peptide 1, peptide YY (PYY), and cholecystokinin (CCK) act opposingly to ghrelin, exerting anorexigenic effects by modulating neurons of hypothalamus nuclei and PVN^[15]^. Interestingly, unlike in genetic leptin deficiencies, knock-out of murine gherlin, of its receptor (growth hormone secretagogue receptor; GHSR) or of ghrelin O-acyltransferase (responsible for prevailingly occurring acetylated ghrelin; GOAT) do not present altered phenotype concerning feeding^[62]^. Similarly, transgenics overexpressing ghrelin and/or ghrelin O-acyltransferase also do not have obesity-related features^[61]^. Ghrelin, as well as leptin, were shown to regulate glucose and glutamate transporters in hypothalamic astrocytes^[70]^. Moreover, the administration of ghrelin increased mitochondrial density in mouse hypothalamic astrocytes^[71]^.

## The obesity-associated hypothalamic inflammation—the role of astrocytes

Obesity, resulting from not only high-fat or highly caloric diets but also genetic dysfunctions (a list of rodent models of obesity is shown in ***Table 1***), is strictly associated with inflammation, both in the periphery and the brain^[72–74]^. As consistently observed in rodent models, the initiation of high-fat diet results, within a few days, in microglia and astroglia activation, manifesting in proinflammatory cytokine release and astrogliosis^[75–76]^. However, exemptions can be found: a high-sucrose diet induced inflammation in the hypothalamus of rats but with no astrogliosis. The neuroinflammation is also present in animal models of genetic obesity^[74,77]^, like the mice with leptin or leptin receptors deficiency. The hypothalamic inflammation associated with excessive weight is also true for humans: in the MRI study, a T2 hyperintensity in the hypothalamus imaging was indicative of inflammation in patients with obesity^[19,78]^. The activation and infiltration of microglia and astrogliosis, associated with hypothalamic inflammation, are contributed by the activation of Toll-like receptors (TLR-4) on both micro- and astroglia, promoting the release of the proinflammatory cytokines, IL-1β, TNF-α and IL-6^[74]^. The transgenic mice with astrocyte-specific knock-out of the myeloid differentiation primary response 88, an adaptor molecule of TLR signaling, presented less pronounced astrogliosis in the hypothalamus than the wild-type controls when treated with a high-fat diet or saturated fatty acids^[79]^. A 12-week of high-fat diet resulted in astrogliosis (GFAP overexpression), concomitant with shortened astrocytic processes and downregulation of astrocyte-specific proteins: glutamate transporters (GLAST and GLT-1) and tight junction forming Cx43^[80]^. However, 12-24 weeks of feeding with a high-fat diet resulted in disruption of myelin integrity in the hypothalamus. This process was mediated by interleukin 33, the level of which was increased in oligodendrocytes and astrocytes^[81]^. The hypothalamic inflammation associated with high-fat diet is a very rapid process—the astrogliosis and increased level of proinflammatory cytokines become observable as soon as 48 hours after starting the diet; therefore, it is proposed that the hypothalamic inflammation is an initiating factor in metabolic dysregulation, *i.a.* insulin and leptin resistance, impaired thermogenesis, associated with obesity^[72,77]^.

**Table 1:** The exemplary rodent models of obesity

Type of the obesity model	Underlying mechanism
Diet-based models	Exceeded calories intake in mice or rats: high-fat diet, high-carbohydrate diet, 'cafeteria' diet
Monogenic obesity models	Spontaneous mutation in the gene coding for leptin (*ob*/*ob* mice) or for leptin receptor (*db*/*db* mice, Zucker fatty rat)
Polygenic obesity models	Selected, susceptible for obesity, inbred strains of mice (New Zealand obese mouse, Tsumura Suzuki obese diabetes mouse) or rats (Wistar Ottawa Karlsburg W Rat, Otsuka Long-Evans Tokushima Fatty Rat)
Surgical models	Lesions of hypothalamus nuclei (*e.g.*, ventromedial or lateral) in mice or rats
For the extensive description of the animals used in obesity studies, please see the reviews^[86,97−98]^.	

The inflammation in the hypothalamus induces endoplasmic reticulum (ER) stress that drives insulin and leptin resistance^[82]^. Insulin resistance is a factor that increases the development of type 2 diabetes, tightly linked with obesity. Since obesity is responsible for over 80% of the risk of developing type 2 diabetes, the term 'diabesity' is being used^[83]^. The hypothalamic inflammation and consequent ER stress lead to persistent activation of the proinflammatory c-Jun N-terminal kinase 1 (JNK1) in AgRP neurons which overactivation, together with co-occurring leptin resistance, results in excessive eating and weight gain. The activity of AgRP and their insulin sensitivity are also disturbed by the upregulation of inflammatory inhibitor nuclear factor κ-B kinase 2^[84]^. Further, signal transducer and activator of transcription 3/suppressor of cytokine signaling 3 and sonic hedgehog pathways may be involved in insulin and leptin resistance^[15,85]^.

The high-fat diet is common, although variable protocols are employed, especially in experimental approach to model obesity in rodents^[86–87]^. In general, the high-fat diet induces obesity, insulin resistance and disturbances in lipid metabolism, as well as depression-like symptoms and cognitive decline in animals fed for several weeks with lard-enriched chow^[76,80,88–89]^.

In obese mice fed with a high-caloric cafeteria diet, the impairment of astrocytic glutamate clearance has been observed. The reduction of GABA tone was a consequence of the overactivation of the metabotropic glutamate receptors by excessive glutamate. The imbalance between excitatory and inhibitory neurotransmission could be rescued by supplementation of antioxidant N-acetylcysteine^[90]^. The high-fat diet also decreased GABA levels in the frontal cortex and hippocampus of rats^[91]^. The disrupted GABAergic inhibitory signaling might underly abnormal feeding behavior.

Also, Sickmann *et al* investigated how type 2 diabetes and obesity affect brain energy metabolism and neurotransmitter systems^[92]^. In Zucker obese and Zucker diabetic fatty rat models, the brain glucose metabolism was lowered, with a more pronounced decrease in the tricarboxylic cycle than in the glycolysis. The reduction in glutamate-glutamine cycle was also observed in the diabetic/obese group in comparison to the lean Zucker rats. Moreover, in the diabetic/obese animals, the glycogen levels were lowered, pointing to the astrocytic involvement as well, despite the relatively lower contribution of the glycolytic pathway. Authors postulate that reduced glutamate/glutamine ratio, revealed by molecular carbon labelling, and resulting decreased glutamatergic neurotransmission, may result from limited ability to glutamine synthesis from glycogen^[92]^. Soontornniyomkij *et al.* however, described increased expression of astrocytic glutamine synthesising enzyme, GS, both in liver and brain of mice fed with high-fat diet^[89]^. The animals consistently presented memory deficits^[89]^. Unfortunately, one cannot distinguish whether the GS overexpression was a compensatory attempt because neither GS activity nor the glutamine levels were measured.

However, the astrocytic activation and neuroinflammation due to the obesity-inducing diets may not always exaggerate pathology, but constitute a response aiming at ameliorating the metabolic disturbances. Accordingly, the high-fat diet may also activate neuroprotective pathways in an attempt to counterbalance the negative outcome. Four weeks of a high-fat diet administered to young mice increased the level of lipids and reduced cytochromes, selectively in astrocytes. Despite that, astrocytic functional domains were enlarged due to augmented branching, and no overexpression of GFAP, GLT-1, or GS was observed. This astrocytic phenotype correlated with better explorative behavior^[93]^. The chromatin remodeling factor, HMG20A, responsible for pancreatic islet beta-cell functional maturation and adaptation to hyperglycemia, was also shown to be expressed in the brain, predominantly in hypothalamic astrocytes. The high-fat or high-glucose diet upregulated HMG20A in astrocytes, which was correlated with increased levels of GFAP and IL-1β. Importantly for clinical relevance, transcription of HMG20A was observed in adipose tissue of diabetic patients with obesity. The silencing of astrocytic HMG20A *in vitro* resulted in the amelioration of the inflammatory and reactive phenotype of astrocytes. Upon HMG20A depletion, astrocytes become more susceptible to apoptosis and the media collected from the culture decreased the viability of neurons. The treatment with ORY1001, a drug mimicking HMG20A, protected mice from the obesity-associated glucose intolerance. Therefore, the data indicate that chronic but low-level inflammation mediated by astrocytic HMG20A may be a neuroprotective response of activated astrocytes^[94]^. Moreover, the intraperitoneally administered IL-6 was found to induce neurogenesis in mice, and prevent from high-fat diet excessive weight gain. The basal expression of IL-6 in the hypothalamus was low but the exogenous IL-6 was able to activate its receptors present on microglia, ependymocytes, endothelial cells, and astrocytes^[95]^.

## Conclusions

**Figure 2:**
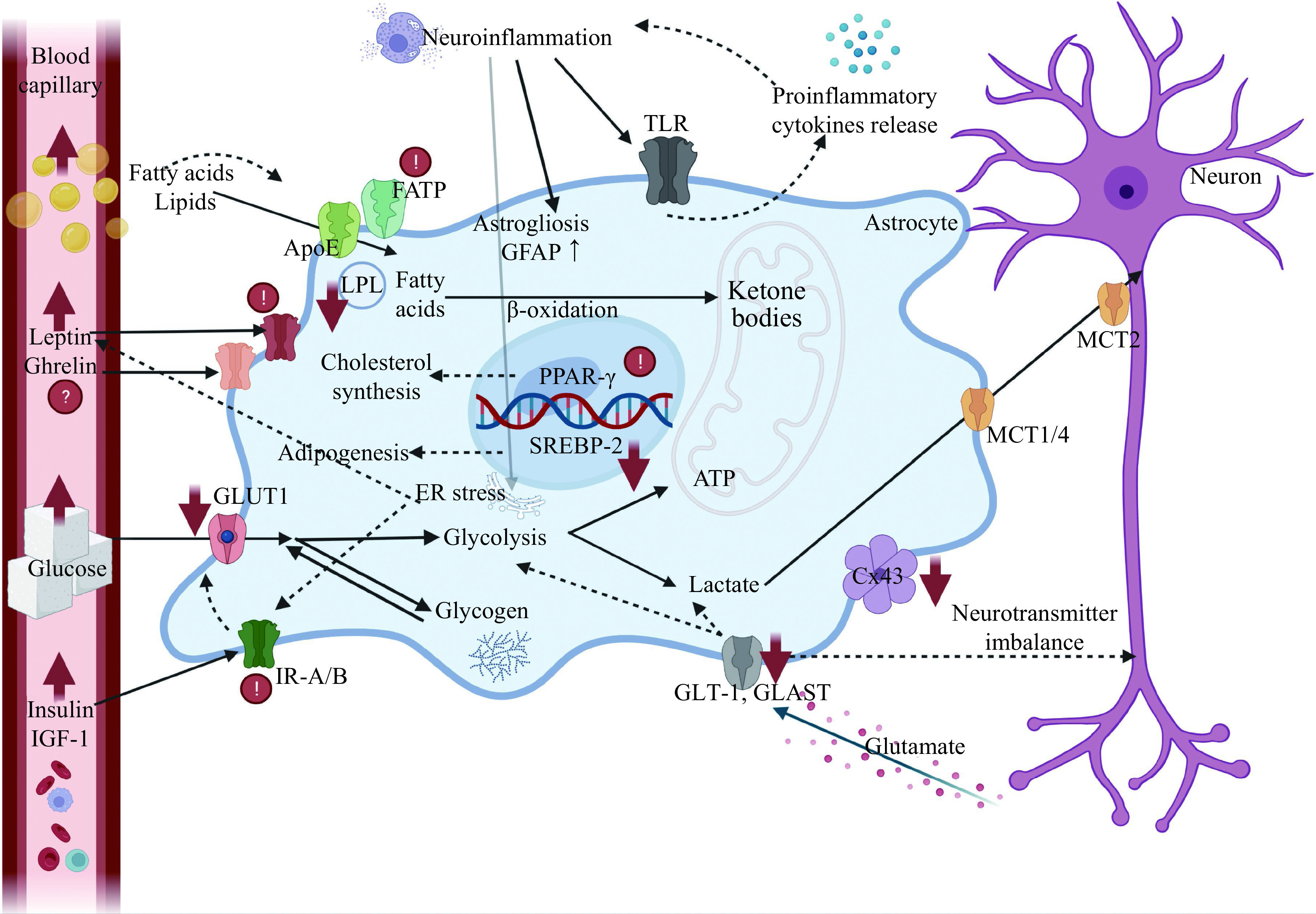
The graphic presentation of metabolic events and molecular signaling mediated by astrocytes, whose impairment may be involved in the pathomechanism of obesity.

The goal of the review was to highlight the scope of astrocytic involvement in the regulation of energy metabolism, as schematically depicted in ***Fig. 2***. Most importantly, the molecular pathways involved in catabolic and anabolic nutrient metabolism are tightly linked with cognitive capabilities. Many of the metabolic disturbances, primarily associated with obesity, like insulin resistance, are common with neurodegenerative processes, *e.g.*, Alzheimer's disease. Therefore, the astrocytes as a target for preventing and treatment of obesity-associated disturbances must be appreciated in the course of research and clinical trials. However, it must be born in mind that most of the current knowledge on pathophysiology of the hypothalamic control over appetite and on modulatory role of astrocytes in this process is derived from rodent studies. What this research does not cover and what is beyond the scope of the present review, is the engagement of the complex systems of reward, emotion, memory, attention, and cognitive control of the human brain^[96]^.
